# Early coordinated rehabilitation in acute phase after hip fracture – a model for increased patient participation

**DOI:** 10.1186/s12877-017-0640-z

**Published:** 2017-10-17

**Authors:** Gillian Asplin, Gunnel Carlsson, Lena Zidén, Gunilla Kjellby-Wendt

**Affiliations:** 1 Institute of Neuroscience and Physiology, The Sahlgrenska Academy, Gothenburg University, Gothenburg, Sweden; 2 Department of Physiotherapy and Occupational Therapy, Sahlgrenska University Hospital, Gothenburg, Sweden

**Keywords:** Hip fracture, Adl, Patient participation, Functional balance, Physical performance

## Abstract

**Background:**

Studies have shown that patients with hip fracture treated in a Comprehensive Geriatric Care (CGC) unit report better results in comparison to orthopaedic care. Furthermore, involving patients in their healthcare by encouraging patient participation can result in better quality of care and improved outcomes. To our knowledge no study has been performed comparing rehabilitation programmes within a CGC unit during the acute phase after hip fracture with focus on improving patients’ perceived participation and subsequent effect on patients’ function.

**Methods:**

A prospective, controlled, intervention performed in a CGC unit and compared with standard care. A total of 126 patients with hip fracture were recruited who were prior to fracture; community dwelling, mobile indoors and independent in personal care. Intervention Group (IG): 63 patients, mean age 82.0 years and Control Group (CG): 63 patients mean age 80.5 years. Intervention: coordinated rehabilitation programme with early onset of patient participation and intensified occupational therapy and physiotherapy after hip fracture surgery. The primary outcome measure was self-reported patient participation at discharge. Secondary outcome measures were: TLS-BasicADL; Bergs Balance Scale (BBS); Falls Efficacy Scale FES(S); Short Physical Performance Battery (SPPB) and Timed Up and Go (TUG) at discharge and 1 month and ADL staircase for instrumental ADL at 1 month.

**Results:**

At discharge a statistically significant greater number of patients in the IG reported higher levels of participation (*p* < 0.05) and independence in lower body hygiene (*p* < 0.05) and dressing (*p* < 0.001). There were however no statistically significant differences at discharge and 1 month between groups in functional balance and confidence, performance measures or risk for falls.

**Conclusion:**

This model of OT and PT coordinated inpatient rehabilitation had a positive effect on patients’ perceived participation in their rehabilitation and ADL at discharge but did not appear to affect level of recovery or risk for future falls at 1 month. A large proportion of patients remained at risk for future falls at 1 month in both groups highlighting the need for continued rehabilitation after discharge.

**Trial registration:**

ClinicalTrials.gov Identifier: NCT03301584 (Retrospectively registered: 4^th^ October 2017).

**Electronic supplementary material:**

The online version of this article (10.1186/s12877-017-0640-z) contains supplementary material, which is available to authorized users.

## Background

Hip fractures are associated with high mortality rates, substantial functional decline and the consequences have been identified as one of the most serious health care problems in elderly people [1]. At present, the age-standardized incidence for females with hip fracture in developed countries is over 150/100000 inhabitants per year, with the highest rates found in Denmark and Sweden at approximately 560/100000. However, due to demographic changes over the coming decades these numbers are expected to increase [2].

A wealth of research to improve outcomes for patients with hip fracture has been performed. Studies include investigating pre-disposing factors affecting recovery [3, 4]; factors associated with increased risk of mortality [4]; effect of lower limb training [5]; recovery of walking ability [6, 7]; predicting risk for future falls [8, 9]; and comparison of orthopaedic versus comprehensive geriatric care (CGC) [10–12].

There is evidence that early admission to a dedicated geriatric unit can reduce mortality and morbidity after hip fracture [13], that an interdisciplinary approach results in better outcomes than standard care [14, 15], and result in improved mobility at 4 and 12 months following fracture [11]. CGC is however not without its limitations. Two recent reviews described how the heterogenous way models of CGC have been organized and put into practice make it difficult to interpret results, compare studies and determine best practice [16, 17]. Rehabilitation research generally includes multiple outcomes, addressing ICF classifications of body function, body structures and activities, however the aspect of patient participation is less commonly studied despite being described as the outcome most important to people with disabilities, their families and society [18]. Encouraging patients with complex health care needs to take a more active role in their health care has shown patients reporting increased motivation and improved outcomes [19], experiencing higher quality of care, with fewer mistakes, and a more positive impression of the health care system [20]. However, the structured nature of care following hip fracture, despite many benefits, has also been described as a factor that can limit patients’ participation due to staff focusing on following care pathways and not allowing time to reflect over and recognise the patients’ personal needs [21].

Factors that may further limit patient participation after hip fracture are impaired functional balance and fear of falling, which not only affect patients’ ability to perform activities of daily living [22], but are considered the most common risk factors for future falls and fractures [23]. Furthermore, reduced balance confidence and impaired functional balance may delay patients’ recovery after hip fracture [24].

At Sahlgrenska University Hospital patients with hip fracture are treated from admission to discharge in a CGC. To our knowledge no study has been performed within a CGC unit to compare rehabilitation programmes during the acute phase after hip fracture with focus on improving patients’ perceived participation and the effect on patients’ function.

The primary aim was therefore to evaluate a modified programme of coordinated inpatient rehabilitation during the acute phase after hip fracture surgery with focus on patients’ perceived participation. Secondary aims were to investigate effect on activities of daily living, functional balance and confidence and physical performance. A further aim was to investigate level of recovery at 1 month follow-up including risk for future falls.

## Methods

### Study design

A prospective, controlled, intervention study. Evaluation of in-patient rehabilitation with follow-up at 1 month post-discharge.

### Setting and participants

This study was performed in a CGC unit, at Sahlgrenska University Hospital in Gothenburg, Sweden comprising three wards, with a total of 78 beds. During September 2013 and May 2014, a convenience sample of 126 patients with hip fracture was recruited. Patients were admitted to one of the three wards depending on available beds. One ward was allocated as the intervention ward and the other two as controls. Inclusion criteria: presenting with acute hip fracture, aged 65 or more, able to speak and understand Swedish, community dwelling pre-fracture, independent walking indoors with or without walking aid and in personal care with exception of bathing/showering. Exclusion criteria: severe drug or alcohol abuse, mental illness or documented cognitive impairment ≤ 8 according to the Short Portable Mental Status Questionnaire (SPMSQ) [25].

### Comprehensive geriatric care (CGC)

All three geriatric wards follow a structured, systematic interdisciplinary geriatric care pathway for hip fracture patients, commencing at admission pre-operatively through to discharge. This follows a fast track approach including; assessment and management of the patient’s somatic and mental health, physical function, ADL ability, social situation, early mobilisation/rehabilitation and early discharge planning. While orthopaedic surgeons are responsible for surgical fixation of the patients’ fracture and routine examination of X-rays after the patient has been weight-bearing, patients are admitted to and cared for throughout their hospital stay by the interdisciplinary team on the geriatric ward.

### Control: usual care treatment

The control group received standard rehabilitation from occupational therapists (OT) and physiotherapists (PT) (Monday to Friday), planned individually and gradually progressed for each patient. Mobilisation with weight-bearing of operated hip was initiated within 24 h of surgery, seven days a week. Patients were provided with a booklet with information about the fracture, operation method, exercise regime and assistive walking and ADL aid available. Information was collected using TLS-BasicADL [26] regarding the patient’s previous levels of physical function and ability to perform activities of daily living (ADL) and assessment of present ability performed as the patient was able. Patients received treatment by a PT on a daily basis (Monday to Friday) including mobilisation and progression of their exercise program, the number of times varied depending on patients’ needs and staff resources. Interdisciplinary team meetings were held twice weekly to discuss progress and future planning. For those patients returning to their own homes, an OT instructed them in the use of ADL aids, and assessed the need for aids in the home environment prior to discharge. All patients received both written and verbal information regarding prevention of falls prior to discharge.

### Intervention

#### Psychological component

##### Enhanced OT and PT collaboration

In addition to standard rehabilitation, focus was placed on promoting patient participation through closer collaboration between the OT, the PT and the patient. Patients were encouraged to take a more active part in and personal responsibility for their training and setting of rehabilitation goals. This involved the OT and PT meeting the patient together within 24 hours postoperatively. They explained their roles in the inpatient rehabilitation process as facilitators to guide the patients in their recovery process whilst making it clear that it was important that the patient felt involved and part of the team.

##### Goal setting using TLS-BasicADL

The TLS-BasicADL protocol was used as in standard practice, however, an additional column for setting goals was added for the purpose of this study, (see Additional file 1). Patients were encouraged, using the TLS-BasicADL protocol, to consider activities that were important to them to be able to perform at discharge. They were invited to answer the following question; “Which activities are important for you to achieve during your inpatient care?” The individual goals were followed up and adapted throughout the hospital stay using the TLS-BasicADL protocol.

##### Supporting patient self-efficacy

To strengthen patient’s self-efficacy by challenging their fear of falling and encouraging patients’ to progress their exercise. This was done under supervision of OT and PT with the aim that patients would gain confidence to take increased responsibility.

#### Physical component

##### Training kit with instructions

To increase activity and encourage patients to take more responsibility for their training out with OT and PT treatment sessions, participants were provided with a training kit consisting of a sliding sheet and leg band to facilitate transfers in/out of the bed, a reaching aid, and stocking aid for training of ADL. Written and photographic instructions were included in the kit. All patients were given self-training exercises to perform daily to suit their level of dependence, adapted and intensified as the patient progressed.

##### Enhanced exercise with protocol

More intensive training of transfers, walking, balance and P-ADL was offered at least 3 times/day by OT and PT (Monday-Friday) from day 2 after surgery compared to control group. OT and PT filled in a training protocol showing when and how often patients received treatment. In addition the patients were encouraged to fill in an exercise diary.

##### Collaboration meetings

Over and above the twice weekly interdisciplinary meetings, the OT and PT met on a daily basis to plan daily training schedules to avoid collision. An additional meeting was held once a week, to further discuss routines concerning collaboration and treatment plans for individual patients. Patients were continually given feedback concerning their progress using the TLS-BasicADL protocol.

Patients were asked daily about adverse reactions to treatment such as increased pain or fatigue. Adverse events were documented in the patient records and treatment was adapted as required.

### Staffing levels and assessors

The OT and PT staffing levels were similar on all three wards, with approximately 0.12 OT and PT per patient. Staff working on the two control wards were informed that the study was in progress, but no information was given regarding the content of the intervention, nor did they treat patients included in the intervention. The two OTs and three PTs who assessed the patients at discharge and one month were not blinded to the intervention but had no treatment association with the study patients.

## Outcome measures

### Demographic characteristics

Pre-fracture baseline data were collected using a specifically designed study questionnaire covering social and living conditions, use of walking aids, frequency of outdoor walks with or without company, and level of social home service/informal help. Data concerning the fracture and other medical conditions were collected from medical records. At discharge, length of hospital stay and discharge destination were reported (Table 1).

**Table 1:** Background data of the patients in the intervention group and control group

Variable		Intervention group *n* = 63	Control group *n* = 63	*P*-value
Age, (years) mean (SD)		82.0 (8.0)	80.5 (7.7)	0.27
Gender, n (%)	Women	47 (75)	49 (78)	0.68
	Men	16 (25)	14 (22)	
Type of fracture, n (%)	Cervical	35 (56)	24 (38)	0.07
	Trochanteric	28 (44)	39 (62)	
Type of surgery, n (%)	LIH/Pinnloc	6 (9)	10 (16)	**0.04**
	Hemiarthroplasty	17 (27)	7 (11)	
	Total hip replacement	10 (16)	8 (13)	
	Plate and screw	8 (13)	11 (17)	
	Intermedullary nail	22 (35)	27 (43)	
ASA score (1–5), n (%)	1–2	42 (67)	37 (59)	0.46
	3–4	21(33)	26 (41)	
General health, n (%)	Good to excellent	35 (56)	37 (59)	0.9
SPMSQ, n (%)	9–10	53 (84)	57 (90)	0.35
Living alone, n (%)		41 (65)	39 (62)	0.8
Housing, n (%)	House	43 (68)	43 (68)	1.0
	Flat	20 (32)	20 (32)	
Assistance from home help services/relative/other, n (%)		35 (56)	36 (57)	1.0
Walking aid prior to fracture indoors n, (%)	None	44 (70)	48 (76)	0.33
	Stick/crutches	6 (9)	3 (5)	
	Rollator/walker	13 (21)	12 (19)	
Walking aid prior to fracture outdoors, n (%)	None	29 (46)	32 (51)	0.81
	Sticks/crutches/nordic	10 (16)	11 (17)	
	Rollator	23 (37)	19 (30)	
	Wheelchair	1 (1)	1 (1)	
Walked outdoors previous month, n (%)	Yes, alone	39 (62)	47 (74)	0.27
	Yes, with company	10 (16)	8 (13)	
	No	14 (22)	8 (13)	
I-ADL, independent n (%)	Cooking	48 (77)	55 (89)	**0.038**
	Cleaning	37 (60)	41 (66)	0.6
	Shopping	41 (66)	39 (63)	0.9
	Transport	50 (81)	47 (76)	0.6
Fall before fracture, n (%)	Yes	25 (40)	25 (40)	1.0
Length of stay, (days) mean (SD)		14.9 (5.9)	13.4 (4.7)	0.13
Discharge destination, n (%)	Home	42 (67)	51 (81)	0.08
	Intermediate rehab	21 (33)	11 (18)	
	Other hospital		1 (1)	

*P*-values for significant differences marked in bold

### Primary outcome

#### Self-rated degree of participation

Self-rated degree of participation in rehabilitation was measured at discharge from hospital. Patients answered 4 questions, specifically formulated for this study, regarding perceived level of participation in their rehabilitation; working together with OT and PT in goal-setting; personal responsibility for their training, and making decisions regarding care and treatment as much as they liked. The questions were answered using a four level scale very high degree, moderate degree, small degree or not at all.

### Secondary outcomes

#### Activities of daily living (ADL)

Ability to perform basic activities of daily living (P-ADL) was assessed using TLS-BasicADL [26]. TLS-BasicADL highlights the patient’s level of independence in basic ADL, comprising of 15 different activities 6 items showing ability to transfer and walk indoors, 7 P-ADL items and 2 additional items negotiating stairs and walking outdoors. Three colour-coded markers indicate level of dependence green = independent, yellow = supervision and red = dependent on physical help of others. TLS-BasicADL does not form a composite score but shows through the colour-coding level of dependence with regard to the patient’s 1) *previous* ability and assistive aids prior to admission to hospital, 2) *present* ability and assistive aids used and 3) *goals* which the patient aims to achieve during inpatient treatment. As the patient’s ability to perform activities changes during in-patient rehabilitation, the colour-coded markers are changed correspondingly. This is done in collaboration with the patient with the aim of promoting increased participation. TLS-BasicADL is also used as a tool for discussion regarding future rehabilitation needs/goals after discharge with the patient. TLS-BasicADL has been shown to have high inter-rater and fair intra-rater reliability [26] and moderate to excellent validity and responsiveness (submitted and under peer review).

Ability to perform instrumental activities of daily living was assessed using IADL items of the ADL-staircase [27].

The ADL staircase is an expansion of the Katz ADL Index [28] of personal activities of daily living, with the addition of four I-ADL items; cooking, shopping, cleaning, and transportation. The ADL staircase uses only two levels, dependent or independent, and can be administered through interview and/or observation. The ADL-staircase has shown good validity and reliability [27], and is considered a stable and clinically relevant tool when used in studies of older people [29, 30].

#### Functional balance

Bergs Balance Scale (BBS) was used to measure functional balance [31] and fall risk [32]. BBS assesses 14 activities of varying difficulty with a scoring range from 0 to 4 (0 unable to perform to 4 able to perform completely) [31]. The item scores are summed giving a score of 0–56, with 56 showing indicating normal functional balance. BBS has shown excellent test-retest reliability and validity [31, 33]. To determine clinical significance, minimal detectable change (MDC) scores described by Donoghue & Stokes [34] were used, ranging from 4 to 7 points depending on baseline score. To discriminate those at risk for falls, a cut-off score of 47 was defined [32].

#### Balance confidence

Balance confidence was measured using the Swedish version of the Falls Efficacy Scale (FES-S) [35]. This version is modified from the original 10-degree scale (1–10) where 1 represents ‘very confident, no fear of falling’ and 10 ‘not confident at all, very afraid of falling’, into an 11-degree scale (0–10) with a reversed answering alternative (0 not confident at all and 10 totally confident). For the purpose of this study the aspect of confidence rather than fear has been assessed. FES-S includes 13 items, comprising three parts, six items measuring self-care, one item stair walking, and six items instrumental activities. The maximum score is 130. Test–retest reliability of the Swedish version of the scale was found to be acceptable by Hellstrom et al. [35].

#### Physical performance

Short Physical Performance Battery (SPPB) [36] consists of three components: standing balance, walking speed - timed 4 m walk, and ability to rise from a chair. The sum of the three components comprises the final SPPB score with a possible range from 0 to 12 (12 indicating the highest degree of lower extremity functioning). According to Perera et al. [37] a small meaningful change is 0.5 and a substantial meaningful change 1.0 point, respectively. For analysis of risk for falls, a score of ≤ 6 is associated with a higher fall rate [9].

The Timed Up and Go (TUG) test measures ability to perform basic everyday movements. TUG assesses total time for standing up from a standard chair, walking 3 m, turning 180 degrees, returning and sitting down. According to recommendations by Podsiadlo and Richardson [38], TUG was performed twice in each test session, one trial and one timed performance, with a brief seated rest in between. The participants were instructed to walk at a comfortable, safe speed. TUG has good inter-rater and intra-rater reliability and is a reliable and valid measure of functional mobility [38]. A TUG score > 24 s at discharge was used for analysis of risk for falls, which is a predictor for falls at 6 months in hip fracture patients [8].

The time schedule for the assessments is shown in Table 2.

**Table 2:** Data collection schedule

Domain	Assessment	Pre-fracture	Post-operatively	At discharge	One month follow-up
Personal ADL	TLS-BasicADL	X	X	X	X
Instrumental ADL	ADL-staircase	X			X
Balance confidence	FES(S)			X	X
Functional balance	BBS			X	X
Functional physical mobility	SPPB, TUG			X	X
Self-rated participation	4-part questionnaire			X	

Pre-fracture information collected retrospectively at hospital

*TLS-BasicADL* Traffic Light System-BasicADL, *FES(S)* Swedish version of Falls Efficacy Scale, *BBS* Bergs Balance Scale, *SPPB* Short Physical Performance Battery, *TUG* Timed ‘Up and Go’ test

## Statistical methods

Based on clinical assumptions and the results of previous studies [39, 40] assuming a power of 80% and ∝ of 0.05, and a difference between groups of 13 points in the Falls Efficacy Scale with SD = 20, a total sample size of *n* = 76 was estimated. With an approximated drop-out rate of 20%, a total sample size of *n* = 92 was necessary. Descriptive statistics are reported as means and standard deviations (SD) or median (min-max) as appropriate. For comparison between the groups at discharge and at 1 month Chi-square was used for analysis of self-rated degree of participation, and P- and I-ADL. Mann-Whitney U Test was used for the analysis of BBS, FES, SPPB and TUG.

For comparison within groups over time Sign test was used for analysis of P-and I-ADL, and Wilcoxon Signed Ranks Test for the analysis of BBS, FES, SPPB and TUG. The level of significance was defined as *p* < 0.05. Analyses were performed using SPSS 21. (SPSS Inc., Chicago, IL, USA).

## Results

Baseline characteristics of the 126 patients recruited to this study are presented in Table 1, with 63 patients in the intervention and control group, respectively. The patients in the intervention group (IG) had an average age of 82.0 years and control group (CG) 80.5 years, with approximately 75% women in both groups (Table 1). No statistically significant differences were found between the groups at baseline apart from type of surgery, with a higher proportion of patients with a hemiarthroplasty in the IG and I-ADL activity of cooking in which the CG was more independent than the IG***.***

**Fig. 1:**
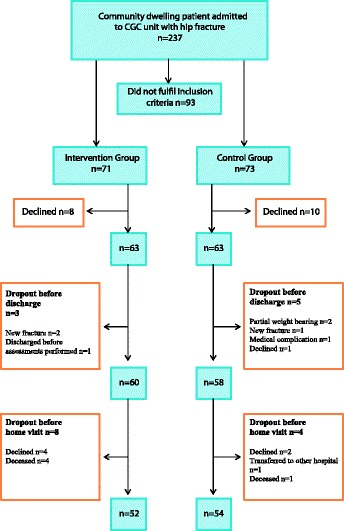
Flow chart showing inclusion and dropout of patients

Of the 237 patients admitted to the unit, 144 patients fulfilled the inclusion criteria. The main reason for exclusion was cognitive impairment and dependency in more ADL activities than just bathing. Eighteen patients declined participation giving a total of 126 patients. There was a further drop-out of 8 patients before discharge, and 12 patients at 1 month, leaving a total of 106 patients completing the follow-up assessment, 52 patients in the IG and 54 in the CG respectively (Fig. 1). Reasons for drop-out prior to discharge included: partial weight-bearing (2), new fracture (3), medical reasons (1), discharged before measurements could be performed (1) and declined (1). Prior to one month follow-up; declined (6), deceased (5), and admitted to hospital (1). This resulted in a 4% mortality and a 16% drop-out rate at 1 month follow-up, (Fig. 1).

## Primary outcome

### Self-rated degree of participation

#### Comparison between groups at discharge

With regard to patients’ perceived participation, statistically significant differences were found between the groups at discharge, with a significantly greater number of patients in the IG reporting higher levels of perceived participation in comparison to the CG, *p* < 0.05 in all four domains (Table 3). Two patients in the IG and one patient in the CG did not complete the questionnaire, leaving a total of 58 and 57 patients in the IG and CG, respectively.

**Table 3:** Degree of perceived participation between the groups at discharge

Participation questions	Degree of participation	Intervention group *n* = 58		Control group *n* = 57		*p*-value
		n	n (%)	n	n (%)	
To what degree do you feel you have participated in your rehabilitation on the ward?	Very high	29	55 (95)	21	44 (77)	*p* = 0.021
	Moderate	26		23		
	Small	3	3 (5)	13	13 (23)	
	Not at all	0		0		
To what degree have you worked together with the OT and PT towards common goals regarding your rehabilitation?	Very high	30	48 (83)	17	36 (63)	*p* = 0.003
	Moderate	18		19		
	Small	10	10 (17)	11	21 (37)	
	Not at all	0		10		
To what degree do you feel you have taken personal responsibility for your rehabilitation?	Very high	37	52 (90)	20	42 (74)	*p* = 0.008
	Moderate	15		22		
	Small	6	6 (10)	11	15 (26)	
	Not at all	0		4		
Have you been involved in making decisions about your care and treatment as much as you wished?	Very high	39	55 (95)	20	45 (79)	*p* = 0.003
	Moderate	16		25		
	Small	2	3 (5)	11	12 (21)	
	Not at all	1		1		

*P*-value indicates significance for dichotomized values

## Secondary outcomes

### Activities of daily living

#### P-ADL (TLS-BasicADL)

##### Comparison between groups at discharge and 1 month

Statistically significant differences were found between the IG and CG in the P-ADL activities of lower body hygiene (*p* = 0.025) and dressing (*p* < 0.001) at discharge, with the IG reporting greater levels of independence (Table 4). By 1 month follow-up, these differences had leveled off between the groups. At 1 month, significant differences were found in the activities of walking up and down stairs and walking outdoors, with a larger proportion of the CG requiring active help than the IG in both activities (Table 4).

**Table 4:** TLS-BasicADL P-ADL, between group differences in levels of dependence at discharge and 1 month

	Discharge			1 month		
TLS-BasicADL Activity	Intervention group	Control group	*p*-value	Intervention group	Control group	*p*-value
Lower body hygiene	*n* = 60	*n* = 58	**0.025**	*n* = 52	*n* = 54	0.734
Independent	41	36		49	50	
Supervision	12	5		0	0	
Active help	7	17		3	4	
Lower body dressing	*n* = 60	*n* = 58	**0.000**	*n* = 52	*n* = 54	0.921
Independent	39	22		41	43	
Supervision	7	1		0	0	
Active help	14	35		11	11	
Stairs	*n* = 33	*n* = 27	0.093	*n* = 46	*n* = 48	**0.009**
Independent	4	8		21	21	
Supervision	21	10		18	8	
Active help	8	9		7	19	
Walking outdoors			n/a as not tested in majority	*n* = 47	*n* = 49	**0.022**
Independent				22	22	
Supervision				16	7	
Active help				9	20	

*P*-values for significant differences marked in bold

#### I-ADL (ADL-staircase)

Concerning I-ADL, no statistically significant differences were reported between the groups in any of the I-ADL items at 1 month follow-up.

##### Comparison within groups at discharge and 1 month

Both groups reported statistically significant improvements in the majority of ADL activities between discharge and 1 month follow-up. Activities where no statistically significant changes were reported included the three activities involving the upper body: upper body hygiene, dressing and eating in which the groups remained highly independent.

**Fig. 2:**
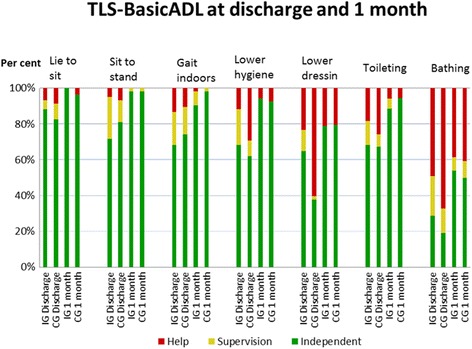
Distribution of degree of dependence in 7 TLS-BasicADL items for both groups at discharge and 1 month

For all participants at 1 month, the activities in which patients were most dependent were walking up and down stairs, and walking outdoors (approximately 60%) followed by showering/bathing (approximately 55%) and lower body dressing (approximately 35%). The distribution of levels of dependence in a selection of seven TLS-BasicADL items can be seen in Fig. 2.

#### Functional balance (BBS), balance confidence (FES-S), and physical performance (SPPB and TUG)

##### Comparison between groups at discharge and 1 month

The results of the outcomes measuring functional balance, balance confidence and physical performance proved to be very similar between the two groups at discharge and 1 month follow-up, with no significant differences between the two groups reported (Table 5).

**Table 5:** Scores of functional balance, balance confidence and physical performance at discharge and 1 month follow up. Comparisons within groups, between groups, differences within groups between discharge and 1 month and change over time

	Comparison of scores at discharge median (range)		*p*-value	Comparison of scores at 1 month median (range)		*p*-value	Comparisons of differences in scores between discharge to 1 month mean (SD)		*p*-value	Comparisons of change over time discharge to 1 month *p*-value	
	IG	CG		IG	CG		IG	CG		IG	CG
Functional balance											
BBS(0–56)	25 (4–52)	28(5–55)	0.868*	38(13–56)	35 (16–56)	0.464*	9.7 (7.3)	7.6 (6.2)	0.122	< 0.000**	< 0.000**
Balance confidence											
FES-S (0–130)	73(7–125)	73(18–130)	0.825*	89(31–130)	90(16–130)	0.981*	19.2(22.2)	17.7(19.2)	0.724	< 0.000**	< 0.000**
Physical performance											
SPPB(0–12)	3.5(0–9)	4 (1–9)	0.533*	5 (1–11)	5 (1–12)	0.268*	2.0(1.7)	1.4 ± 1.9	0.083	< 0.000**	< 0.000**
TUG (sec)	32 (12–114)	28.5 (10–120)	0.852*	20(10–173)	22 (8–95)	0.808*	−11.2(22.2)	−12.5(19.5)	0.755	< 0.000**	< 0.000**

*IG* intervention group, *CG* control group

*Mann Whitney U Test

**Wilcoxon Signed Ranks Test

##### Comparison within groups at discharge and 1 month

Statistically significant improvements were reported in both groups for all measures between discharge and 1 month follow-up. Both groups showed clinically significant differences in BBS and SPPB, with improvements exceeding the recognised MDC scores (Table 5).

### Number of falls reported at 1 month

A total of ten patients reported having fallen since discharge, two patients in the IG and eight in the CG, these results were however not statistically significant.

#### Risk for falls (BBS, SPPB, TUG)

##### Comparison between groups at discharge and 1 month

With regard to BBS the majority of the patients in both groups scored considerably lower than the cut-off score of ≤ 47, discriminating those at risk for falls. At discharge 93 and 95% in the IG and CG, respectively, had failed to reach above 47 points, while the proportion of patients at risk decreased at 1 month, 75 and 78% remained at risk for IG and CG, respectively.

For SPPB, 91 and 90% in IG and CG, respectively, failed to score above the cut-off value of 6 for fall risk at discharge, which improved to 69 and 66%, respectively, at 1 month follow-up.

The results of the TUG scores revealed that 64 and 68% in IG and CG, respectively, scored above 24 s indicating risk of falling at discharge, which improved to 36 and 42%, respectively, at 1 month (Table 6).

**Table 6:** Risk for falls at discharge and 1 month

	Discharge		1 month	
Outcome and cut-off score	Intervention Group n (%)	Control Group n (%)	Intervention Group n (%)	Control Group n (%)
BBS ≤ 47	54 (95)	54 (95)	42 (81)	44 (85)
SPPB ≤ 6	53 (91)	51 (90)	36 (69)	35 (66)
TUG > 24 s	36 (64)	37 (68)	19 (36)	22 (42)

*BBS* Bergs Balance Scale, *SPPB* Short Physical Performance Measure, *TUG* Timed up and Go

##### Adverse events

No adverse side effects from the intervention were reported, suggesting that older persons following hip fracture surgery both tolerate and benefit from a more coordinated and intensive rehabilitation compared to standard care.

## Discussion

This study shows that by modifying existing CGC in-patient rehabilitation routines and intensity, whilst retaining existing staffing levels, positive results concerning patient participation and recovery of ADL can be obtained. There is a lack of studies investigating patient participation during acute phase after hip fracture. However, our physical performance outcomes are similar to those reported by Prestmo et al. who compared orthopedic care and CGC [11]. They found no difference between groups at 1 month concerning physical performance [11]. In our study both groups improved beyond the recommended MDC levels for both balance and physical performance measures between discharge and 1 month. However, despite these improvements, approximately 40–80% of patients remained at risk for future falls at 1 month.

### Strengths and limitations

#### Strengths

The difficulties in comparing studies due to the varying models of orthogeriatric care in practice and the heterogeneity of patients with hip fracture are well-known [41]. However, a strength of this particular study is that we have compared rehabilitation models of care within a well-established geriatric unit, specialized in care of older persons with hip fracture. To decrease the heterogeneity of the study population, we chose to examine previously relatively high functioning older adults. No changes were made in the levels of OT or PT staffing on the 3 wards and were comparable to staffing levels in the orthogeriatric unit described in the study performed by Prestmo et al. [11].

A further strength is the use of recommended and recognized outcomes to measure balance and physical function, which could facilitate comparisons of results with future studies. BBS has recently been recommended as one of two outcomes to measure standing balance for research and practice in adult populations [42]. SPPB and TUG are also commonly used in studies of patients with a hip fracture and community dwelling older adults [11, 32, 43–47]. There was to our knowledge, no established questionnaire evaluating perceived patient participation for this patient group, and for this purpose questions were therefore constructed by the authors of the study. These questions were tested in 10 patients prior to starting inclusion in the study, and according to patient feedback minor revisions were made.

#### Limitations

Although a randomized controlled study would have strengthened this trial, it was not possible due to different admission routines depending on day of the week and time of admission. Our power calculation initially showed that we needed 92 patients. However we did not stratify for gender, which resulted in a maldistribution towards the end of the inclusion process. We therefore chose to continue to include participants until a balance was reached between women and men, which resulted in a total of 126 participants.

We recognise that there were more patients with cervical fractures and ASA 1–2 in IG which in theory should mean they be less compromised early post-operatively. However our clinical observation was that patients in the IG were in fact less able than those in the CG. This may have contributed to more IG patients being discharged to an intermediary rehab unit and not directly home. Even if there were no statistically significant differences, the IG was 1.5 years older and CG was more independent in cooking and walking outdoors, suggesting a slightly higher level of pre-fracture function. While we need to be cautious, this could partly explain a higher proportion of CG being discharged to own home.

The differences found in patient participation we believe, are a result of the more coordinated approach by OT and PT, which incorporated recommendations described by Sahlsten et al. [19]. While it may be argued that these recommendations are included in standard care, the intervention placed greater focus on the OT and PT together forming a relationship with the patient earlier and adapting/progressing treatment more pro-actively in collaboration with their needs and wishes. TLS-BasicADL was used in this process as an instrument to inform and discuss patients’ previous and present ADL ability, and provide a visual aid for discussing meaningful goals e.g. being able to transfer independently in order to go to the toilet without calling on assistance*.* The IG received a greater level of individual support by the OT and PT when exercising, at least three times/day. The IG was also encouraged to be more actively involved in their rehabilitation, both during, and between, treatment sessions.

We also believe that the improvements in ADL are the result of the earlier ADL training, more intensive treatment and closer collaboration with the OT and PT. By 1 month follow-up these differences in ADL had disappeared, which may be attributed to the normal recovery process with mobility improving as swelling and pain subsides. Two activities were however shown to be statistically significantly better in the IG: climbing stairs and walking outdoors. The number of patients fully independent in these two activities was similar between the groups. However, a greater number in the IG required only supervision in both activities in contrast to active physical help in the CG. The reason for this is however not clear, and the results should therefore be treated with caution.

Balance confidence (FES-S) scores improved between discharge and 1 month, but no statistically significant differences were found between the groups. The intervention implemented in this study stopped at discharge, with both groups continuing to follow local routine. While it is recognised that continued rehabilitation is beneficial for persons following hip fracture [3, 48] the rehabilitation services provided after discharge vary considerably in Sweden and were out with the scope of this study. The number of patients who received treatment from a PT between discharge and one month was similar between the groups, but the content and intensity were unknown. A study performed by Zidén et al. [48], reported a higher degree of balance confidence in a similar patient group receiving hospital-based home rehabilitation compared to conventional treatment 1 month after discharge. Our results are comparable to these of the control group that received conventional care who scored 85.5 in (FES-S), but remain considerably lower than the 117 reported by the home rehabilitation group.

Our extended aim was to describe level of recovery at 1 month with regard to ADL function, functional balance and physical performance outcomes according to cut-off scores for risk for falls. Regaining independence in ADL and mobility is the main goal of in-patient rehabilitation [41]. The three activities in which both groups showed least recovery were: showering/bathing, stairs and walking outdoors, with less than half of the patients being independent in these activities at one month. We recommend that these activities should be included in routine rehabilitation programmes and offered to patients after discharge in order for patients to regain optimal levels of function. At present there is no structured rehabilitation programme for this patient group after discharge from acute hospital care.

The total number of patients reporting having fallen between discharge and 1 month follow-up was approximately one in ten however it would have been of interest to follow these participants over a longer period in order to explore how many patients were readmitted to hospital due to falls. A recent review [49], reported that exercise programmes that included challenging balance exercises, performed at least 3 h/wk can reduce rates of falls. However, no evidence was found to support exercise as a single intervention for patients recently discharged from hospital. This is in line with the study performed by Rapp et al. who reported that this may have minor benefit for patients during the first weeks after discharge from hospital. This is in line with the study performed by Rapp et al. who reported that exercise alone may have minor benefits for patients during the first weeks after discharge from hospital, and emphasise the importance of good discharge planning in order to optimize the support given to patients in their home environment to prevent future falls [50].

The results of our study add to the knowledge concerning rehabilitation and outcomes after hip fracture surgery. Further research is however required to enhance our understanding of what is important and of value to patients during acute in-patient rehabilitation in order to better equip them for discharge from hospital into the community. It is also important to communicate the continuing risk for falls to the primary care and community rehabilitation services to highlight and motivate the need for early rehabilitation post-discharge, in order to improve services, outcomes and to prevent future falls.

## Conclusion

This model of OT and PT coordinated inpatient rehabilitation had a positive effect on patients’ perceived participation in their rehabilitation and ADL but did not appear to affect level of recovery or risk for future falls at 1 month. A large proportion of patients in both groups remained at risk for future falls at 1 month highlighting the need for continued rehabilitation.

## Additional file

Additional file 1: Example of TLS-BasicADL protocol. (PDF 272 kb)

## Abbreviations

ADL: Activities of daily living
ASA: American society anesthesiologists score
BBS: Bergs balance scale
CG: Control group
CGC: Comprehensive geriatric care
FES(S): Falls efficacy scale (Swedish version)
I-ADL: Instrumental activities of daily living
IG: Intervention group
MDC: Minimal detectable change
OT: Occupational therapist
P-ADL: Personal activities of daily living
PT: Physiotherapist
SPMSQ: Short portable mental status questionnaire
SPPB: Short physical performance battery
TLS-BasicADL: Traffic light system – BasicADL
TUG: Timed up and go
