# Supplementary material for: Early coordinated rehabilitation in acute phase after hip fracture – a model for increased patient participation
Source: BMC Geriatr. 2017 Oct 17;17:240. doi: 10.1186/s12877-017-0640-z (PMC5646112; doi:10.1186/s12877-017-0640-z)
Supplement: Additional file 1: — Example of TLS-BasicADL protocol. (PDF 272 kb) [file 12877_2017_640_MOESM1_ESM.pdf]

TLS-BasicADL

Name: Peter

PRIOR		PRESENT	Nr people	Aids	GOALS
	LYING to SITTING		1	band	
	SITTING to LYING		1	band	
	SIT to STAND			rollator	
	BED to CHAIR			rollator	
none	GAIT/WHEELCHAIR INDOORS		1	rollator 50 m	rollator
	HYGIENE UPPER BODY				
	HYGIENE LOWER BODY		1		
	SHOWERING		1	shower seat	
	DRESSING UPPER BODY				
	DRESSING LOWER BODY		1	stocking aid/reacher	
	TOILETING		1		
	EATING				
3 flights stick	STAIRS		1	1flight with stick	stick
rollator	GAIT/WHEELCHAIR OUTDOORS			not tested	rollator

Latest assessment

Physiotherapist: Karin Occupational therapist: Anna

6/8

Gillian Asplin, Fysioterapi, SU/Mölndal 2016-02-17
